# Complete Genome Sequence and Manual Reannotation of Mycobacterium avium subsp. *paratuberculosis* Strain DSM 44135

**DOI:** 10.1128/MRA.00711-20

**Published:** 2020-08-13

**Authors:** Ralph Goethe, Tina Basler, Thorsten Meissner, Elke Goethe, Cathrin Spröer, Jolantha Swiderski, Gerald-F. Gerlach, Siegfried Weiss, Michael Jarek, Boyke Bunk

**Affiliations:** a Institute for Microbiology, Department of Infectious Diseases, University of Veterinary Medicine Hannover, Hannover, Germany; b Leibniz Institute DSMZ, German Collection of Microorganisms and Cell Cultures, Braunschweig, Germany; c German Centre of Infection Research, Partner Site Hannover-Braunschweig, Braunschweig, Germany; d Department of Molecular Immunology, Helmholtz Centre for Infection Research, Braunschweig, Germany; e Institute of Immunology, Medical School Hannover, Hannover, Germany; f Genome Analytics, Helmholtz Centre for Infection Research, Braunschweig, Germany; University of Maryland School of Medicine

## Abstract

Here, we report the complete genome sequence of the Mycobacterium avium subsp. *paratuberculosis* reference strain DSM 44135, amended with a manual genome reannotation. The strain was originally described as *M. paratuberculosis* strain 6783. It was isolated from feces from a dairy cow in northern Germany.

## ANNOUNCEMENT

Mycobacterium avium subsp. *paratuberculosis* causes paratuberculosis, also called Johne’s disease, a chronic progressive and fatal enteritis of ruminants (1). Here, we provide the complete genome sequence of the M. avium subsp. *paratuberculosis* reference strain DSM 44135. This strain was originally described by Jark et al. (2) and was later characterized by IS*900* restriction fragment length polymorphism typing as a cattle type strain (3).

M. avium subsp. *paratuberculosis* strain DSM 44135 was provided by the Leibniz Institute DSMZ. Different preparations of genomic DNA from cultures derived from the same original authentic certified DSMZ sample were used for sequencing. For all preparations, mycobacteria were cultured to late-exponential-growth phase at 37°C in Middlebrook 7H9 medium with glycerol, oleic acid, albumin, dextrose, and mycobactin. DNA was isolated using a Genomic-tip 100/G (Qiagen, Hilden, Germany) and quality checked using a Bioanalyzer (Agilent). The SMRTbell template library was prepared according to the instructions provided by Pacific Biosciences (PacBio, Menlo Park, CA, USA), using the procedure and checklist for >10-kb template preparation and sequencing. Genomic DNA was sheared using g-TUBEs from Covaris (Woburn, MA, USA) according to the manufacturer’s instructions. Size selection for the removal of small fragments was performed using 0.375× AMPure beads. Briefly, for preparation of 10-kb libraries, ∼10 μg genomic DNA was end repaired and ligated overnight to hairpin adapters using components of the DNA polymerase binding kit P4 (PacBio). Reactions were carried out according to the manufacturer’s instructions. The SMRTbell template was treated with exonuclease to remove incompletely formed reaction products. Conditions for annealing of sequencing primers and binding of polymerase to the purified SMRTbell template were assessed by the calculator in RS Remote, followed by single-molecule real-time (SMRT) sequencing on the PacBio RS II platform, taking one 120-min movie for each SMRT cell. In total, four SMRT cells were analyzed, resulting in 180,823 reads with a mean (filtered) length of 3,949 bp. Libraries for Illumina sequencing were prepared using the TruSeq DNA library preparation kit and sequenced on the GAII (11,909,029 reads of 2 × 76 bp) and MiSeq (16,145,000 reads of 2 × 251 bp) platforms (Illumina, San Diego, CA, USA). Quality control of Illumina reads was performed using FastQC (https://www.bioinformatics.babraham.ac.uk/projects/fastqc). Long-read genome assembly was performed with the RS HGAP Assembly v3 protocol included in SMRT Portal v2.0.1, applying a target genome size of 5 Mbp. Quality control, in terms of filtering with a minimum subread length of 500 bp and minimum polymerase read quality of 0.8, was performed with SMRT Portal v2.0.1. The resulting chromosomal contig was circularized. In particular, artificial redundancies at the ends were removed and adjusted to *dnaA*. Identification of *dnaA* and redundancies was carried out using BLAST. Circularization and rotation to *dnaA* were performed with the genomecirculator.jar tool (https://github.com/boykebunk/genomefinish). Error correction was performed by mapping of Illumina short reads onto the completed genome using CLC Genomics Workbench v7.0.3 with subsequent determination of a new consensus sequence. Genome annotation was based on Prokka v1.8 (4) with subsequent manual curation. Default parameters were used for all software unless otherwise specified. In this case, the annotation of the whole genome was improved gene by gene by subsequently comparing amino acid sequences to the following databases: the KEGG Orthology database, the TIGRFAMs database, Escherichia coli K-12 reference proteomes, Swiss-Prot characterized proteins, and the Conserved Domain Database. Product names, gene symbols, EC numbers, and coding sequence (CDS) start sites were curated.

The final circular chromosome consists of 4,839,032 bp with 4,563 predicted genes and 4,430 CDSs, of which 245 carried signal peptide sequences. The GC content was determined to be 69.3%. The genome harbors 3 rRNA genes and 58 tRNA genes. The average sequencing depths were 121× and 1,378× for long and short reads, respectively.

### Data availability.

This genome sequence has been deposited in NCBI GenBank under accession number CP053068. The version described in this paper is the first version, CP053068.1. Raw sequence reads have been submitted to the NCBI SRA under the accession number of the corresponding BioProject, PRJNA628963, including SRX8293683, SRX8293684, SRX8293685, and SRX8293686 (PacBio RS II), SRX8353769 (Illumina GAII), and SRX8353770 (Illumina MiSeq).
